# Using ^157^Gd doped carbon and ^157^GdF4 nanoparticles in proton-targeted therapy for effectiveness enhancement and thermal neutron reduction: a simulation study

**DOI:** 10.1038/s41598-022-22429-0

**Published:** 2022-10-18

**Authors:** Farshid Tabbakh, Narayan S. Hosmane, Suffian M. Tajudin, Amir-Hossein Ghorashi, Nader Morshedian

**Affiliations:** 1 Plasma and Nuclear Fusion Research School, Nuclear Science and Technology Research Institute, Tehran, 14155-1339 Iran; 2 Department of Chemistry and Biochemistry, Northern Illinois University, DeKalb, IL 60115-2862 USA; 3 Faculty of Health Sciences, Universiti Sultan Zainal Abidin, Kuala Terengganu, Terengganu Malaysia

**Keywords:** Nuclear physics, Biological physics, Radiotherapy, Targeted therapies

## Abstract

There are two major problems in proton therapy. (1) In comparison with the gamma-ray therapy, proton therapy has only ~ 10% greater biological effectiveness, and (2) the risk of the secondary neutrons in proton therapy is another unsolved problem. In this report, the increase of biological effectiveness in proton therapy has been evaluated with better performance than ^11^B in the presence of two proposed nanomaterials of ^157^GdF4 and ^157^Gd doped carbon with the thermal neutron reduction due to the presence of ^157^Gd isotope. The present study is based on the microanalysis calculations using GEANT4 Monte Carlo tool and GEANT4-DNA package for the strand breaks measurement. It was found that the proposed method will increase the effectiveness corresponding to the alpha particles by more than 100% and also, potentially will decrease the thermal neutrons fluence, significantly. Also, in this work, a discussion is presented on a significant contribution of the secondary alpha particles in total effectiveness in proton therapy.

## Introduction

In radiotherapy, there are some physical advantages in using the proton beam rather than the gamma-ray, such as the beam shaping capability to minimize the dose leakage to the healthy tissue^1^. But still this modality is facing some serious challenges. One of these challenges is releasing the secondary neutrons during the treatment and the risk of the second cancer developing^2,3^. On the other hand, the biological effectiveness in proton therapy is only 10 percent greater than that of the^60^Co gamma-ray therapy^1,4,5^ beside its higher price in comparing to the other modalities^6^. The vanishing of the neutron risk and increasing the effectiveness are the motivations of the present research work.

As several previous researches proposing to improve the biological effectiveness by proton-targeted therapy^7–10^, in which the specific agents have been sent into the tumor site and the interaction between the incident protons and the target will generate more secondary particles (particularly the alpha particles) which potentially induce extra dose to tumor. Accordingly, the use of boron enriched nanomaterials has been suggested to increase the high LET alpha particles through the fusion interaction of proton-^11^B^9^. However, many researches were verifying the impact of proton-boron fusion therapy method and found different results^11–18^.

In our present report, the use of gadolinium-doped carbon nanoparticles and gadolinium fluoride (GdF4) nanoparticles was proposed as the candidate targets in proton-targeted therapy with the purpose of increasing the biological effectiveness and also reducing the secondary neutrons. Carbon and fluorine have considerable cross-section for capturing the incoming protons in tumor to produce the high LET alpha particles;^19^

$$p+{}^{12}C\to 3\alpha +p \,\,(400 mb),$$

$$p+{}^{9}F\to \alpha +{}^{16}O\,\, (500 mb),$$

and also isotope of^157^Gd has a huge cross-section for capturing the thermal neutrons;

$$n+{}^{157}Gd\to \gamma +{}^{158}Gd \,\,(250000 b).$$

Accordingly, the biological impacts and neutron risk reduction related to these proposed compounds have been analyzed in the present study. The novelty of these materials over using^11^B in Proton-Boron Fusion Therapy (P-BFT)^9^ is that when considering the use of nanocomponents in delivering the agents, there are more atoms of carbon or GdF4 in each nanoparticle targets, while in P-BFT method, there is only 1–2% wt of^11^B in each nano-target^20,21^ which causes a very small probability of proton-boron interactions. Also, boron concentration in tumor is 100 ppm^22,23^ but GdF4 is the agent already being used in bimodal imaging and demonstrates lower toxicity in vitro and in vivo and hence can be concentrated up to 200 ppm^24–26^. Moreover,^157^Gd can be used with high concentration up to 3000 ppm^27–30^ in tumor which potentially will vanish the risk of the second cancer by the thermal neutrons.

In present study, the micro-dosimetry calculations were performed using GEANT4 Monte Carlo tools^31–33^ and the measurement of the strand breaks were performed utilizing the GEANT4-DNA package^34–36^.

## Methods

In the present study, the enhancement of effectiveness was evaluated through the increase of the secondary alpha particles when the proton impinging the two proposed targets. To perform a realistic condition, the clinical proton beam was emitted to the tumor at the given depth and the proton spectrum was obtained. The obtained proton spectra were considered as the input energy of the proton impinging the proposed targets. Also, the calculation related to the neutron capture were performed based on the incoming clinical proton beam to the tumor at the given depth as well. First, the clinical proton beam setup for the present study has been explained. Then, the target’s geometry and materials have been described. It is emphasized that the nano-targets were bombarded by protons with the energies according to the energy spectra inside the tumor. After that, the calculation methods for assessing the biological impacts and also the neutron reduction have been presented.

### Clinical proton beam

The tumor site (1 cm side cube) was placed between two depths of 2–3 cm as proximal and distal end, respectively. The primary proton beam with Spread-Out Bragg’s Peak (SOBP) provides a uniform distribution of proton dose along the tumor depth. Therefore, using the primary beam with SOBP, the initial beam has energy range of 50–60 MeV corresponding to the Bragg’s positions of 2 cm and 3 cm, respectively. When the tumor was irradiated by the clinical beam, the protons energy inside the tumor has the energy spectra from thermal up to 32 MeV. This energy spectrum inside the tumor volume was considered as the input for the protons energy impinging the micro-volume cell.

### Target’s geometry and materials

**Figure 1:**
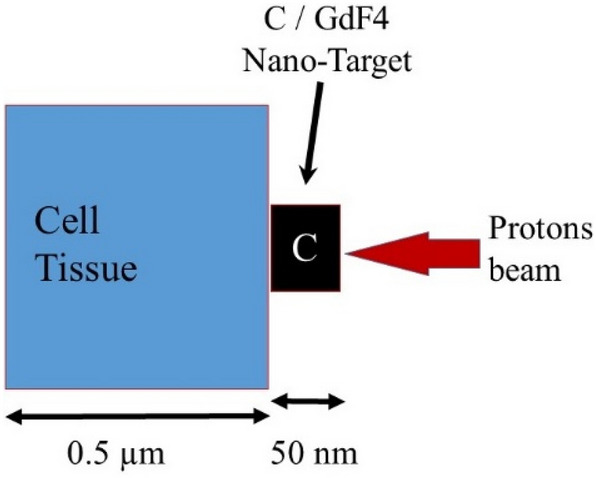
The geometry of the target in simulation. The cell tissue of 0.5 µm side cube, as a small segment of the tumor site, has been irradiated by protons having the energy spectra. The C or GdF4 nano-target, 50 nm side cube was placed between the incident protons and the cancerous tissue to increase the number of the secondary alpha particles.

For the present micro-dosimetry study, the nano-size agent was placed between the incident proton and the micro-size volume of cancerous tissue as illustrated in Fig. 1. The irradiated cancerous tissue was considered 0.5 µm side cube which is comparable to the dimension of the chromatin fiber similar to the geometrical data reported by Bernal and Liendo^37^. The incident proton will interact with the target atoms and produce the secondary alpha particles through the nuclear interactions; ${}^{12}C\left(p,3\alpha \right)p$ and ${}^{9}F\left(p,\alpha \right){}^{16}O$.

The density and elemental mass fraction have been presented in Table 1. This table also presents the related data for carbon nanoparticles and GdF4 nanoparticles. The nano-target in the form of 50 nm side cube as was placed between the tissue and the incident protons. It should be noted that in search of the most proper size of the nano-target, the thickness of smaller than 50 nm was leading to the smaller interactions with the protons and thus the lower number of produced alpha particles. When the thickness is increased to almost 100 nm, no significant variation was detected in producing the alpha particles.

**Table 1:** The cell tissue used in present calculations consists of H, O and C with the mass fraction presented in this table according to Ref.^38^. The target materials with fractional mass also have been presented.

Material	Mass fraction	Density (g/cm^3^)
Tissue^38^	Hydrogen (10%), Oxygen (70%), Carbon (20%)	1.1
Carbon (C)	Carbon (100%)	2.2
Gadolinium Fluoride (GdF4)	Gadolinium (68%), Fluor (32%)	7.1

### Calculation methods for biological effectiveness

In the present work, the impact of the mentioned nano-targets in enhancing effectiveness has been assessed by two different analyses. The total Linear Energy Transfer (LET) and dose enhancement and also, using the GEANT4-DNA package to calculate the number of the strand breaks induced by the secondary alpha particles. Both analyses were based on the energy spectrum of the produced alpha particles.

#### Method-1: the LET and dose calculation

As the first step, after irradiating the cell by protons, the spectrum of the secondary alpha particles in terms of energy (and the LET) has been calculated in the presence and absence of the nano-target. In each energy bin, the increase of the particle’s fluence will increase the resulted dose to the surrounding tissue. Thus, the proposed method is based on increasing the number of the secondary alpha particles to increase the total dose. The total dose can be obtained from integrating the $fluence({E}_{i})\times LET({E}_{i})$ over the energy bins^39^;

1 $${Dose}_{Tot}(Gy)=\sum_{i}fluence({E}_{i})\times LET({E}_{i})\times 1.6{\times 10}^{-13},$$

where in, $fluence({E}_{i})$ and $LET({E}_{i})$ are corresponding to the *i*th energy bin.

The LET(*E*_*i*_) was extracted from the data reported by Sato et al.^40,41^ and ICRP (publication *103*)^42^. Additionally, we have presented the RBE value of the alpha particles as a function of LET necessary for the Results and Discussions section which was based on the RBE-LET relationship for 10% surviving fraction reported by Tracy et al.^43^. Table 2 represents the RBE and LET reference values corresponding to energy groups of the alpha particles.

**Table 2:** The LET as a function of energy of the alpha particles has been presented based on the data Refs.^40,41^. Also, the RBE as a function of the LET has been presented in this table according to Ref.^43^ for 10% surviving fraction.

*E*_*i*_ (MeV)	LET(*E*_*i*_)^40^ (keV/μm)	RBE^43^
1	200	2.4
2	140	3.7
3	130	4.5
4	110	4.3
5	100	4.3
6	90	4.3
7	80	4
8	70	3.5
9	60	3.3
10	55	3.2
11	54	3.1
12	53	3

#### Method-2: the strand breaks using GEANT4-DNA

In addition to the above analysis, which was based on the dose enhancement, the increased number of the DNA damages with the use of nano-targets have been calculated by the GEANT4-DNA package^34–36^. When one alpha particle with energies of 1–12 MeV (*E*_*i*_ = 1–12 MeV) impinging the cell, single strand breaks (SSB)s and double strand breaks (DSB)s was calculated based on the alpha particle’s $fluence({E}_{i})$. The total number of the SSBs and DSBs (per incident proton) can be obtained as;

$${SSB}_{tot}=\sum_{i}\left[fluence({E}_{i})\times SSB({E}_{i})\right]$$

and

2 $${DSB}_{tot}=\sum_{i}\left[fluence({E}_{i})\times DSB({E}_{i})\right]$$

#### Calculation methods for neutron capture by^157^Gd

The reduction of the neutrons risk by adding the^157^Gd has been evaluated according to a macroscopic study. For this purpose, the initial proton beam with SOBP passing through the tissue toward the tumor placed at the depth of 2 cm as described in “Clinical proton beam” subsection. The calculations were performed *with* and *without* gadolinium. The tumor was considered made of tissue (Table 1). In capturing the neutron,^157^Gd has two advantages over^10^B; the concentration of greater value than that of^10^B and also, the higher cross-section for capturing the thermal neutrons, (250000 *b*) which is several orders of magnitude greater than that of^10^B, (4000 *b*)^44,45^.

For evaluating the thermal neutron reduction, we have calculated the thermal fluences in tumor *with* and *without*^157^Gd. As an important note, it is emphasized that the use of^157^Gd in the present study is to capture the secondary neutrons for the purpose of reducing the neutron’s risk. Therefore, the proposed method is completely different from the NCEPT method^46^ in using^10^B and^157^Gd for the enhancement of effectiveness in proton therapy.

### GEANT4 physics list

Geant4 is an open source Monte Carlo toolkit for simulation of the passage of particles through matter, developed by CERN^27,28^. In simulation by Geant4 it is required to determine the physics related to the problem. The following physics lists have been used for the proton inelastic reactions, elastic interactions and the electromagnetic physics, respectively. The proton inelastic interactions in our simulations were addressed by *G4HadronPhysicsQGSP_BIC_HP* in which, QGSP (Quark Gluon String Pre-compound) is the basic physics list below 10 GeV for the nuclear inelastic interactions and BIC (Binary Ion Cascade) for nuclear de-excitations. Term HP refers to the High-Precision cross sections. Also, the elastic interactions were addressed by *G4HadronElasticPhysicsHP,* the High-Precision hadronic elastic physics model. For the electromagnetic interactions, *G4EmStandardPhysics* option4 was used, similar to the work performed by Incerti et al.^47^. For estimating the number of the SSBs and DSBs when the alpha particle (with different energies) passes through the tissue, we have utilized the GEANT4-DNA ruling by *G4EmDNAPhysics* physics list^34–36^.

For neutrons inelastic and capture interactions, *G4NeutronHPCapture* and *G4NeutronHPinelastic* were used, respectively. The number of events in our simulation was 10^8^, since we have found that in micro and nano-dosimetry simulations, performing for 10^8^ events lead to the maximum precision for the results.

## Results

**Figure 2:**
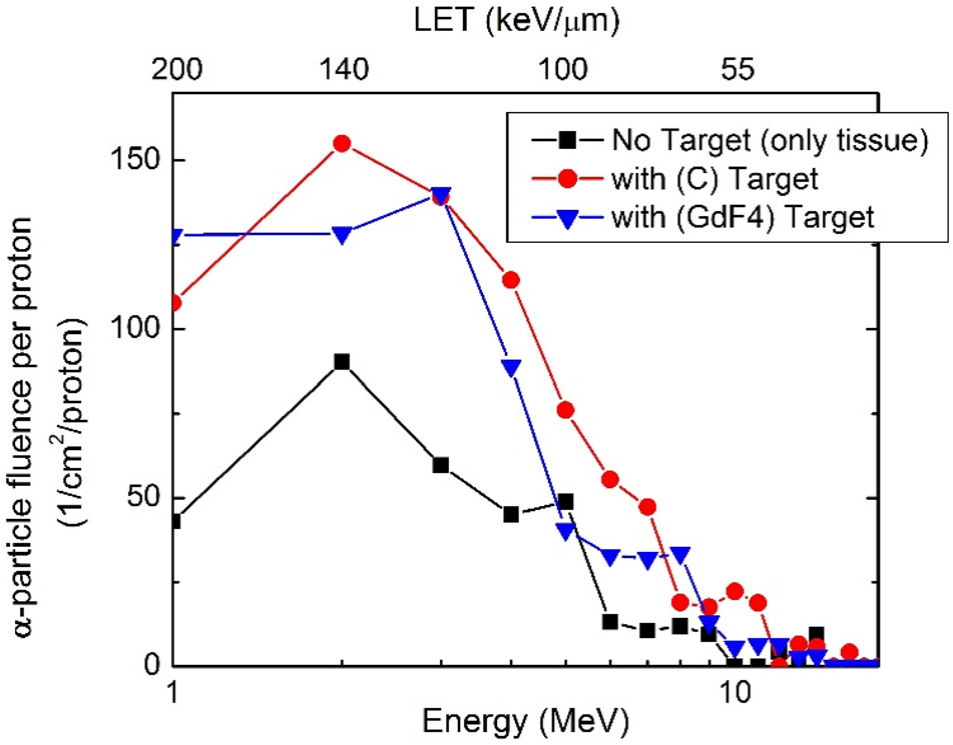
The energy spectrum (and the LET spectrum) of the alpha particles when the cell was irradiated by protons in the presence of one nano-target of C and GdF4 (colored lines) and also without nano-targets (black line). The fluences corresponding to the highest LET particles (> 100 keV/µm) have been increased more significantly than the low LET particles when compared to without nano-target spectrum (black-line).

As indicated above, the present analyses were based on the increasing amount of the secondary alpha particles and, thus the effectiveness was increased when the target was added. Therefore, we have simulated the spectra of the secondary alpha particles in the presence and absence of the targets. The results are depicted in Fig. 2 showing the variation of the alpha particles fluence in terms of energy and LET according to the energy-LET relationship reported by Stao et al.^40,41^. This figure shows a considerable increase of the alpha particles when the two proposed targets are included. The energy range of these extra alpha particles are important for biological assessments. From Table 2 and also according to the LET-RBE relationship^48,49^, the highest LET is not necessarily representing the highest RBE value, but this figure shows most of the increased alpha particles having energies of 1–6 MeV corresponding to the highest LETs of 110–200 ($\frac{keV}{\mu m}$) and almost to the highest RBE. Since the assessment of the enhancement of effectiveness in terms of RBE is relatively complicated and depends on several parameters, such as the different organ’s radiosensitivity, instead of direct calculation of RBE, we have performed our effectiveness evaluations based on two parameters; the total dose and also the number of the strand breaks as the single-valued metrics to describe the biological impact of the increased alpha particles.

### Results of effectiveness enhancement

The dose related to the alpha particles, corresponding to *with* and *without* nano-targets, have been calculated from the LET-fluence in Fig. 2 and the Eq. (1). The obtained results presented in Table 3 showing the alpha particles dose (Gy/proton) has been enhanced 110% and 100% corresponding to carbon and GdF4, respectively (almost one order of magnitude), comparing to without target irradiation or conventional proton therapy.

**Table 3:** The calculated total dose related to the α-particles without nano-target (no agent) and in presence of one nano-target of C and GdF4. As it is shown, the dose has been enhanced almost 100% when one nano-target is included.

Agent	Total dose (Gy/proton)	Enhancement (%)
No Agent	7 × 10^–9^	–
C	1.5 × 10^–8^	110%
GdF4	1.4 × 10^–8^	100%

**Figure 3:**
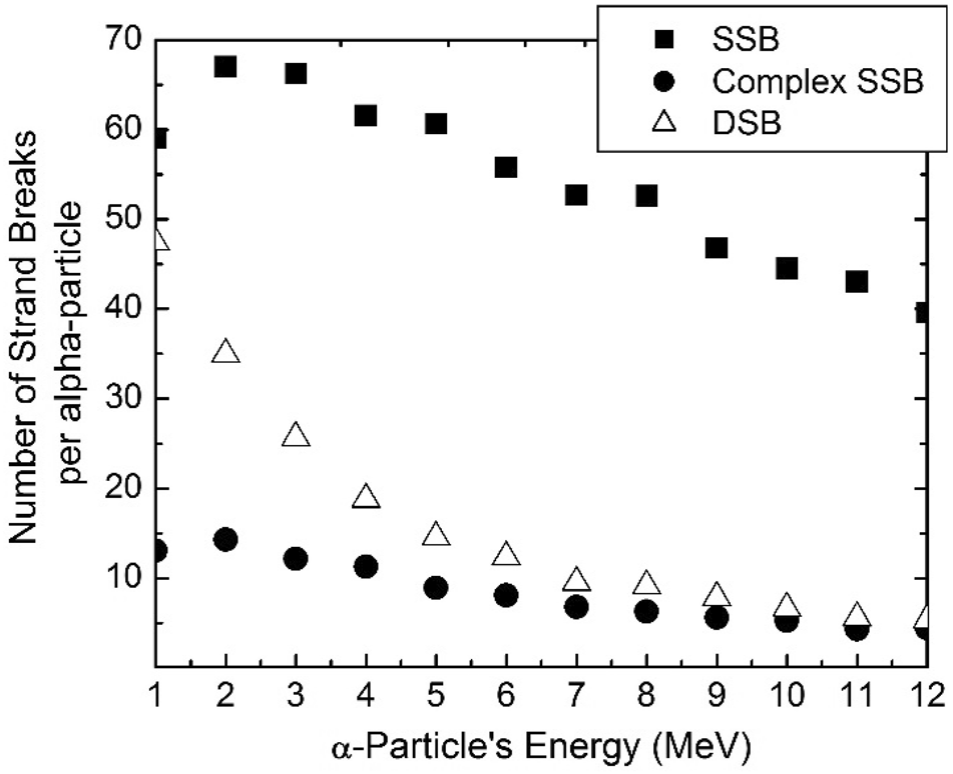
The number of strand breaks, SSBs and DSBs produced per alpha particle as a function of particle’s energy calculated by GEANT4-DNA for the water phantom 0.5 μm side cube. As it is shown, the SSB and DSB curves have maximum values at 2 MeV and 1 MeV, respectively, which correspond to the highest LET values (Table 2).

To evaluate the cell eradication impact in the presence of proposed targets, first the number of the (SSB)s and (DSB)s induced by one single alpha particle with different energies (from 1 to 12 MeV) have been simulated by GEANT4-DNA. Figure 3 shows the variation of the SSB, complex SSB and DSB for one alpha particle in terms of the particle’s energy which is in agreement with the result reported by Tracy, et al.^38^. Considering the different fluences related to different energies (from Fig. 2), the total damages per one incident particle can be obtained by Eq. (2). As it has been shown in Table 4, we have found that the inclusion of the proposed targets indicated that the number of the SSBs has been enhanced 124% and 100% corresponding to C and GdF4 nano-targets, respectively, and the number of the DSBs also has been raised 120% and 90%, respectively.

**Table 4:** The total numbers of SSB and DSB have been calculated according to the particle spectrum corresponding to without nano-target (no agent) and with one nano-target of C and GdF4. When one C nano-target is added, the SSB and DSB values increase to 124% and 120%, respectively. When one GdF4 nano-target is added, the resulted increases are 100% and 90%, respectively.

Agent	(SSB)/proton	Damage enhancement (%)	(DSB)/proton	Damage enhancement (%)
No agent	2.1 × 10^4^	–	8.8 × 10^3^	–
C	4.7 × 10^4^	124%	1.92 × 10^4^	120%
GdF4	4.0 × 10^4^	100%	1.7 × 10^4^	90%

Accordingly, the two proposed materials caused the effectiveness of the alpha particles to be enhanced significantly with a relatively greater effectiveness by using carbon comparing with that of GdF4 target. Moreover, the non-toxicity of carbon is another advantage of using this target because it can be concentrated in tumor with a greater amount than other candidate targets, such as^11^B (with maximum 100 ppm) in P-BFT method^9^. However, the diagnostic applications of GdF4^23–25^ could be considered as another potential candidate in proton-targeted therapy and imaging, simultaneously.

### Important discussion on significant role of the alpha particles in proton therapy

Here, we have presented the explanation about the importance of increaring the secondary alpha particles in proton therapy as an important parameter for increasing the number of the cell damage despite their lower contribution in effectiveness than of the primary protons.

In proton therapy, the biological effects from the proton beam is significantly greater than effects from the produced secondary particles such as the secondary proton, deuteron,^3^He,^4^He (alpha particles) and other heavy charged particles. When proton beam passes through the tissue, ~ 20% of the protons will contribute in nuclear interaction leading to a lower fluences related to the produced secondary particles^3,4^ which would lead to a much lower impact regarding the secondary produced particles. But, the impact of the alpha particles are different from other secondary particles and is not negligible, especially in a microscopic point of view. As it has been discussed and explained by Paganetti^50^, the microdosimetric measurements show that the alpha particles play an important role in radiation quality and dose-averaged value. Since their contribution is scaled by a high-RBE value, the impact of the alpha particles in calculation of their biological effectiveness is remarkable and should be strongly considered. In this regard, several researchers such as Kraft^51^ and Cirrone^52^ have addressed the important contribution of the alpha particles in the subcellular level as well. Accordingly, in proton therapy the increase of the secondary alpha particles will lead to the increase of the cell eradication process and effectiveness which reveal the significance of using the proper agents, such as those proposed in this study for increasing the number of alpha particles.

### Results of the neutron reduction

To estimate the reduction of the neutrons by^157^Gd, as described above, the analysis has been performed in the macroscopic volume for the tumor (1 cm side cube) placed at the depth of 2 cm from the tissue’s surface. First, the fluence of the thermal neutron inside the tumor was calculated and then,^157^Gd has been added to the tumor with high concentration around several thousand ppm^27–30^ to evaluate the increase of capturing the secondary neutrons by gadolinium and thus, reducing the risk of the neutrons for developing the secondary tumors.

**Figure 4:**
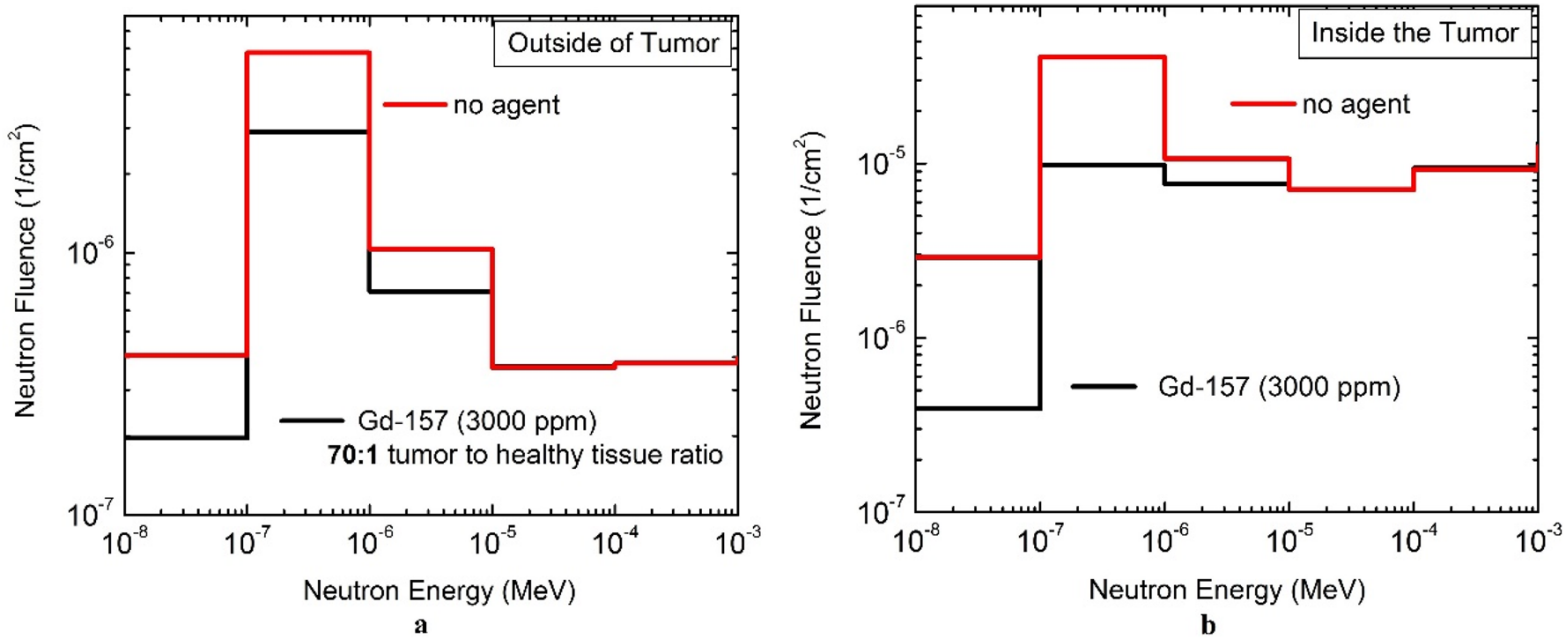
The variation of the secondary neutrons (from thermal up to 1 keV) in proton therapy when 3000 ppm of gadolinium-157 has been concentrated in tumor with 70:1 tumor to healthy tissue ratio. (**a**) Depicts more that 50% decrease in thermal to epithermal neutron fluences outside of tumor (in healthy tissue) and (**b**) shows more than one order of magnitude decrease in neutron inside of tumor.

As described earlier, the proton irradiation (by SOBP proton beam) was performed *with* and *without*^157^Gd and the related neutron fluence have been calculated. In our study, 3000 ppm of^157^Gd was used as suggested by Morrison^27^ and Safavi^46^, while Tucomitsu^53^ suggested the concentration of 6000 ppm in tumor. Figure 4 reveals the amount of neutron reduction in the presence of 3000 ppm with 70:1 tumor to healthy tissue concentration ratio based on the literature^46^. As it is shown, for outside of tumor (in healthy tissue) shown in Fig. 4a the thermal up to epithermal neutrons have been reduced almost 50% and for the neutron inside the tumor shown in Fig. 4b, almost one order of magnitude decrease has been achieved.

Table 5 also presents the amount of neutron reduction when 3000 ppm of^157^Gd is added to the tumor. Based on the results, the thermal and epithermal fluences per proton have been reduced from 2.0 × 10^–6^ to 3.0 × 10^–7^ for inside tumor and from 4.0 × 10^–7^ to 2.0 × 10^–7^ for healthy tissue region with 70:1 tumor to healthy tissue concentration ratio. Consequently, the proposed method of using gadolinium showed the potential of vanishing the low energy neutrons (thermal up to 1 keV) for the future trials.

**Table 5:** Reduction amount of the secondary neutrons in energy range of thermal to 1 keV. The fluence (per proton) inside and outside the tumor has been measured and compared to the fluence when 3000 ppm of gadolinium-157 has been added.

Agent	Thermal fluence per proton	
	Healthy tissue	Inside tumor
Whitout^157^Gd	4 × 10^–7^	2.0 × 10^–6^
^157^Gd (3000 ppm)^27–30,46^	2 × 10^–7^	3.0 × 10^–7^

## Conclusions

In the present study, the enhancement of effectiveness in proton targeted therapy was investigated through the increase of the high-LET alpha particles by proposing two candidate targets, the^157^Gd doped carbon and gadolinium fluoride (^157^GdF4). The reason of considering carbon and fluorine as the candidate targets is their considerable cross-section with proton for the production of high-LET alpha particles. Also,^157^Gd has been proposed as a potential target for capturing the thermal neutron in order to reduce the neutron risk in proton therapy. The results were obtained by GEANT4 and GEANT4-DNA for the related calculations. According to the results, the presence of the proposed nano-targets will increase the biological effectiveness more than 100% by producing alpha particles. In addition, the thermal to epithermal neutron fluence was reduced around one order of magnitude by adding 3000 ppm of^157^Gd showing its potential for vanishing the neutrons risk in proton therapy.

## Data Availability

The datasets generated and/or analyzed during the current study are available from the corresponding author.
